# Gender Differences in The Factors associated with Hypertension in Non-Diabetic Saudi Adults—A Cross-Sectional Study

**DOI:** 10.3390/ijerph182111371

**Published:** 2021-10-29

**Authors:** Rajaa Al-Raddadi, Jawaher Al-Ahmadi, Suhad Bahijri, Ghada M. Ajabnoor, Hanan Jambi, Sumia Enani, Basmah Medhat Eldakhakhny, Lubna Alsheikh, Anwar Borai, Jaakko Tuomilehto

**Affiliations:** 1 Saudi Diabetes Research Group, King Fahd Medical Research Center, King Abdulaziz University, Jeddah 3270, Saudi Arabia; rmsalharbi@kau.edu.sa (R.A.-R.); jalahamade@kau.edu.sa (J.A.-A.); gajabnour@kau.edu.sa (G.M.A.); hjambi@kau.edu.sa (H.J.); senani@kau.edu.sa (S.E.); beldakhakhny@kau.edu.sa (B.M.E.); lloo86@hotmail.com (L.A.); Boraiaa@ngha.med.sa (A.B.); jotuomilehto@gmail.com (J.T.); 2 Food, Nutrition and Lifestyle Research Unit, King Fahd for Medical Research Centre, King Abdulaziz University, Jeddah 22252, Saudi Arabia; 3 Department of Community Medicine, Faculty of Medicine, King Abdulaziz University, Jeddah 22252, Saudi Arabia; 4 Department of Family Medicine, Faculty of Medicine, King Abdulaziz University, Jeddah 22252, Saudi Arabia; 5 Department of Clinical Biochemistry, Faculty of Medicine, King Abdulaziz University, Jeddah 22252, Saudi Arabia; 6 Department of Food and Nutrition, Faculty of Human Sciences and Design, King Abdulaziz University, Jeddah 3270, Saudi Arabia; 7 King Abdullah International Medical Research Center (KAIMRC), King Saud bin Abdulaziz University for Health Sciences (KSAU-HS), College of Medicine, King Abdulaziz Medical City, Jeddah 22384, Saudi Arabia; 8 Department of Public Health, University of Helsinki, 00014 Helsinki, Finland; 9 Public Health Promotion Unit, Finnish Institute for Health and Welfare, 00271 Helsinki, Finland

**Keywords:** hypertension, prehypertension, lifestyle, obesity

## Abstract

The association between lifestyle practices, obesity and increased BP are under-investigated. We aimed to investigate this association to identify the factors associated with hypertension and prehypertension in Saudis. Non-diabetic adults were recruited from public healthcare centers using a cross-sectional design. Recruits were interviewed using a predesigned questionnaire. Weight, height, waist circumference (WC), hip circumference (HC), neck circumference (NC) and BP were measured. The variables were analyzed by comparing the prehypertensive and hypertensive groups with the normotensive group. A total of 1334 adults were included. The study found that 47.2% of men and 24.7% of women were prehypertensive, and 15.1% of men and 14.4% of women were hypertensive. High BMI, WC, NC, and WC: HC ratios were associated with an increased risk of prehypertension and hypertension in men and women. Low physical activity was associated with an increased risk of elevated BP in men, while sleep duration of ≤6 h and sitting for ≥4 h were associated with increased risk in women. Women from central Asia, southeast Asia, and those of mixed origin had a higher prevalence of hypertension compared to those from Arabian tribes. In conclusion, prehypertension and hypertension increase with age and obesity. Gender differences were apparent in the association between several lifestyle practices and prehypertension or hypertension among various ethnic/racial groups.

## 1. Introduction

Hypertension is a strong risk factor for cardiovascular disorders and has been found to be the main predictor of cardiovascular morbidity and mortality [1,2]. Indeed, a study published in 2008 reported that hypertension alone was the cause of 13.5% of the total global premature deaths (7.6 million), as well as 54% of strokes, and 47% of incidences of ischemic heart disease [3].

Moreover, various epidemiological studies on different populations and ethnicities have shown that higher levels of BP, including levels in the prehypertension state, are widely associated with increased risk of fatal and nonfatal cardiovascular events [4,5,6]. The associated medical cost and human capital loss increases the burden to the economy [7,8].

In Saudi Arabia, the reported prevalence of hypertension has been increasing over the years [9,10,11,12,13,14], with the most recent study reporting it to be 31.4% [15]. Furthermore, hypertension was classified as the leading risk factor for death among Saudis in 2010 [16]. Unfortunately, as noted in other studies [17], hypertension was undetected in a large percentage of the Saudi population during a 2013 national survey, and 57.8% of studied people with hypertension were undiagnosed [14]. In view of these reports, steps should be taken to increase awareness among the Saudi population to control the trend of increasing hypertension-related mortality. In addition, healthcare professionals should be aware of the risk factor associated with high blood pressure for this population.

Increased blood pressure (BP) is often a consequence of obesity [18]. However, other disease states, as well as some lifestyle practices, may lead to hypertension [19,20]. The noted increase in the prevalence of hypertension in Saudi Arabia might be attributed to lifestyle changes related to urbanization and the adoption of dietary eating habits that are likely to result in hypertension, as well as the increasing prevalence of obesity [21]. The relationship between lifestyle practices, different measures of obesity, and hypertension has not been fully investigated in the Kingdom of Saudi Arabia. Furthermore, no studies of the effect of ethnic origin on the prevalence of hypertension have been conducted in Saudi Arabia, even though the Saudi people descend from different races, and racial disparity in hypertension and hypertension-related outcomes has long been acknowledged [22]. Therefore, we aimed to investigate the factors associated with prehypertension and hypertension among persons of various racial/ethnic groups in Saudi Arabia.

## 2. Materials and Methods

A cross-sectional design was employed to conduct this study, which is part of a more extensive study intended to validate a “dysglycemia risk score” for Saudi people that has been outlined fully previously [23]. A total of 1477 adults were recruited. However, after excluding those found to be diabetic and those with missing data, 1334 adults were included in the study. Data were collected by trained medical students. Anthropometric measurements (weight, height, waist circumference, hip circumference, and neck circumference) and BP were taken using standardized techniques [24]. Recruits were interviewed for medical history and demographic and lifestyle characteristics using a predesigned questionnaire. Blood samples were obtained while fasting for an estimation of a glucose and lipid profile. Another sample was taken one hour after the ingestion of a 50 g of oral glucose load for the estimation of glucose and glycated hemoglobin (HbA1c) [25].

The definitions of hypertension and prehypertension followed those outlined in the Joint National Committee on Prevention, Detection, Evaluation and Treatment of High BP (JNC) VII report [24]. Prehypertension was defined as systolic blood pressure: 120 to 139 mm Hg and/or diastolic blood pressure 85 to 89 mm Hg, and hypertension was defined as systolic ≥ 140, and/or diastolic ≥ 90 mm Hg or taking blood pressure-lowering drug treatment.

### Statistical Methods

Statistical analysis was carried out using SPSS, version 21. Descriptive statistics were calculated for all measured and estimated parameters and are presented as mean ± standard deviation (SD) for continuous variables and as frequency (percentages) for categorical variables. Demographic, lifestyle and clinical factors of hypertension were analyzed by comparing those of prehypertensive and hypertensive participants and those of normotensive group. Factors with continuous variables were analyzed using ANOVA and independent t-test, while those with categorical variables were analyzed using Chi-square test or Fisher’s exact test, as appropriate.

Multiple logistic regression analysis was used to adjust for age. Unadjusted and adjusted Odds Ratio (OR) with its 95% Confidence Interval (CI) for the factors associated with prehypertension and hypertension are presented. Statistical significance was assigned at *p* < 0.05.

## 3. Results

A total 1334 adults were included in the current study, the mean age and age groups according to BP status of men and women are presented in Table 1.

A high percentage of the participants were either prehypertensive (47.2% of men and 24.7% of women) or hypertensive (15.1% of men and 14.4% of women). Only 43.7% of men, and 56.3% of women were found to have normal blood pressure. A higher percentage of people were prehypertensive, or hypertensive due to elevated systolic blood pressure. More than 93% of Prehypertensive people, and even a considerable percentage of people with hypertension (about 64%) were unaware of their condition.

The mean age of hypertensive men and women was significantly higher than the mean age of prehypertensive and normotensive corresponding groups. However, the mean age of prehypertensive women was significantly higher than the mean age of the normotensive group. Only 56.3% (95% CI: 46.6–65.6) of hypertensive men, and 34.1% (95% CI: 24.2–45.2) of women were <35 years old, as shown in Table 1.

Lifestyle, demographic and anthropometric characteristics in the hypertensive, prehypertensive and non-hypertensive study groups are presented in Table 2 and Table 3.

As shown in Table 3, general obesity, abdominal obesity, and upper-torso obesity were significantly higher among people with elevated blood pressure. The mean BMI of hypertensive men and women was significantly higher than the mean BMI of both prehypertensive and normotensive men and women. In addition, the means of the prehypertensive groups were also significantly higher than that of the corresponding normotensive groups. Only 25.9% (95% CI: 18.1–35.0) of hypertensive men, and 25.9% (95% CI: 18.1–35.0) of hypertensive women had normal WC. Similar findings were noted when waist to height ratio, waist to hip ratio, and neck circumference results were compared. Ethnicity, physical activity, and, to some extent, sleep duration were also different when comparing the three groups. However, gender differences were obvious in the case of these factors. Men descending from southeast Asia and those from African tribes had a higher percentage of hypertension than expected according to the total distribution of ethnicities, although the difference did not reach statistical significance. In the case of women, those descending from central Asia, southeast Asia, and mixed origin showed a higher percentage of hypertension than expected, while those from Arabic tribes had a significantly lower percentage (*p* < 0.01). The effect of physical activity was statistically apparent in men, but not in women, with a higher percentage than expected of physically inactive men having normal blood pressure.

In contrast, women with a sleep duration of ≤6 h had a lower percentage of normal blood pressure than prehypertensive women. No significant difference was found between the three groups of men or women with respect to sitting hours, smoking habits, nor daily intake of fruit and vegetables. Very few people reported eating the recommended minimum amount of three portions daily of fruit and vegetables. Therefore, at least one portion daily was used in our analysis.

After adjusting for the effect of age, the results are presented in Table 4 for prehypertension and hypertension and Table 5 for hypertension alone.

After adjusting for age, all measures of obesity (BMI, abdominal obesity level 2, neck circumference, elevated waist to hip and to height ratios) were found to be associated with elevated blood pressure in men and women. In addition, low physical activity was found to be associated with elevated blood pressure in men, while sleep duration of ≤6 h and sitting for ≥4 h were associated with elevated blood pressure in women. Ethnicity, and smoking were not associated with elevated blood pressure, Table 4.

Similarly, after adjusting for age, all measures of obesity (BMI, abdominal obesity level 2, neck circumference, elevated waist to hip and waist to height ratios) were also found to be associated with hypertension in both men and women. In addition, descending from central Asia, and, to a lesser extent, from southeast Asia and being of mixed origin were associated with hypertension among women. However, none of other investigated lifestyle factors showed a significant association with hypertension, as shown in Table 5.

## 4. Discussion

In this study, we aimed to investigate the association between lifestyle practices, ethnic origin, as well as different measures of obesity and higher than desirable blood pressure to identify the factors associated with hypertension and prehypertension among Saudi men and women. To avoid bias and ensure a good representation of the Saudi population, the sample was randomly collected from attendees of PHCCs in Jeddah whose inhabitants cover all socioeconomic sectors and ethnicities living in Saudi Arabia [26]. Both prehypertension and hypertension were common in our study population, which is close to previously published prevalence data for hypertension on Saudi populations, although it should be kept in mind that our study included non-diabetic persons only while previous studies included all people [12,14,27]. Only one of the previous studies, however, reported the prevalence of prehypertension as 66.1%, 48.1% and 54.9% in men, women and all people, respectively [27]. The latter study was conducted in Alkharj city which is located in central Saudi Arabia, south of the capital Riyadh, with a population descending from Arabic tribes, and composed of mixed urban (military and civilian), rural, and adjacent nomadic communities [27]. In view of the reported racial disparity in the prevalence of hypertension and prehypertension [22], our study is the first to present the prevalence of prehypertension in the largest city on the western side of Saudi Arabia, the population of which reflects the different ethnicities living in the country as noted from our results. The lower prevalence in our study could be due to the exclusion of diabetic people from our analysis.

The prevalence of prehypertension was studied in other Gulf countries. In Oman, the prevalence of prehypertension in a population sampled from a national screening program of chronic non communicable diseases in primary health care institutions was reported to be 45% [28], while in prediabetic adults the prevalence was reported to be 54.1% [29]. In a pilot study among healthy adults in the United Arab Emirates (UAE), the prevalence of prehypertension was 42.9% among men and 16.9% among women [30], which is close to our own results. In Kuwait [31], and Bahrain [32], the prevalence of prehypertension in college students was reported to be 39% and 37%, respectively, emphasizing the effect of age on estimated prevalence.

An earlier national study [14] investigated the association of various sociodemographic and lifestyle factors and hypertension only. Saudi Arabia is a large country, with each of its regions having its own characteristic mixture of ethnicities, dietary and lifestyle practices. The national study was carried out on the population as a whole without distinguishing between the different regions. Furthermore, the study included people with diabetes. This might have led to incorrect conclusions due to the effect of diabetes on diet and lifestyle practices.

Therefore, our study is the first in the Saudi kingdom to investigate the association between lifestyle factors and increased blood pressure (prehypertension and hypertension) among non-diabetic people in a city of mixed ethnicities.

Many modifiable and non-modifiable factors have previously been reported to increase the risk of high blood pressure. Age, ethnicity, and gender are all non-modifiable factors. Aging and being male were found to increase the prevalence of elevated blood pressure, as prehypertension and hypertension in our study in accordance with previous Saudi studies [12,14,27], as well as studies in other populations [28,29,30,33,34,35,36,37]. Therefore, data were analyzed for men and women separately, and age was adjusted for when calculating the association and risk assessment with various previously known risk factors.

It was interesting to note that the effect of ethnicity was apparent among female participants, but not significant among men, during initial analysis of data. Racial differences in the risk of pre-hypertension and hypertension have long been reported, even for people living in the same country [22,38]. After adjusting for age, women descending from central Asia, and, to a lesser extent, from southeast Asia and of mixed origin had a significantly increased risk of hypertension [OR (CI): 11.54 (2.76–48.18), 3.51 (1.22–10.13), 3.24 (1.04–10.08) respectively].

Obesity, measured by different indices, has long been recognized as an independent and important risk factor for the development of hypertension in different populations [39,40,41,42,43]. After adjusting for age, general obesity (BMI ≥ 30 kg/m^2^) was associated with prehypertension and hypertension to a greater extent for men than women (OR 3.36 for prehypertension, and 5.38 for hypertension among men, and 2.65 for prehypertension, and 3.18 for hypertension among women). Measures of abdominal obesity (level 2 high WC, high WC: height, and WC: hip ratios), as well as neck circumference, were associated with an increased risk of prehypertension and hypertension among both men and women to a similar extent. Obesity was associated with hypertension in an earlier Saudi study [12] and a more recent national survey [14]. Gender difference in the association between anthropometric measurements and hypertension was reported earlier in a large Chinese study on older adults, indicating the need to develop gender-specific strategies for the male and female elderly in the primary and secondary prevention of hypertension [44].

The gender difference in risk factors became more apparent when the effects of lifestyle factors were investigated.

Prospective epidemiological evidence has indicated that sedentary behavior, defined as any waking behavior characterized by an energy expenditure ≤ 1.5 metabolic equivalents while in a sitting or reclining posture [45], including office work, is associated with increased risk of various clinical and population health problems, such as type 2 diabetes and cardiovascular disease, hence increasing the risk of mortality [46,47]. On the other hand, increased physical activity has been reported to be associated with lower blood pressure [48,49]. In this study, prehypertension, but not hypertension as such, was associated with ≥4 h/day of sitting among women and with low physical activity (<the recommended 30 min/day–5 days/week) among men. The association between elevated blood pressure and sedentary behavior has been reported in various studies carried out on people of different ethnicities and age groups, including children [50,51,52,53,54,55,56]. Moreover, a recent systematic review and meta-analysis examined the associations between time spent in sedentary behaviors and blood pressure in both adults and children, concluding that for each hourly increase in self-reported sedentary behavior, there was an associated small increase in systolic and diastolic blood pressure of 0.06 (95% CI, 0.01–0.11) and 0.20 (95% CI, 0.10–0.29) mm Hg, respectively. In addition, there was a 2% elevation in the risk for hypertension (odds ratio, 1.02; 95% CI, 1.003–1.03) for each hourly increase in sedentary behavior [57]. Decreased physical activity and sedentary behavior have been linked to becoming overweight and obese [58,59,60]. Indeed, sedentary behavior has been reported to be more independently and strongly associated with becoming overweight and obese than physical activity [59,61]. The association between sedentary behavior and obesity might explain the noted association with increased blood pressure in our study.

Another gender difference in the effect of lifestyle practices was noted when the association between sleeping hours and blood pressure was examined. Prehypertension was found to be associated with a shorter sleep duration of ≤6 **h** among women, but not men. In the CARDIA sleep study, higher systolic and diastolic blood pressure were reported to be associated with a short sleep duration and lower sleep maintenance (the percent of the time during the sleep period spent sleeping) after adjustments for confounders and the exclusion of participants on antihypertensive drug treatments [62]. Moreover, extensive evidence for the association between a short sleep duration and prehypertension and hypertension was found in a recent review which included both cross-sectional and longitudinal epidemiologic studies [63]. However, two Saudi studies linked hypertension to a longer sleep duration [64,65]. Both studies investigated the association between sleep duration and various factors and health characteristics in Saudi adults without excluding people with diabetes. In contrast, our approach was different, as we investigated the association between lifestyle factors, including sleep duration, and high blood pressure in a non-diabetic population.

The first study investigated the association between sleeping hours and BMI, hypertension, and hyperglycemia, concluding that people with hypertension slept for >8 h/night [64]. This study was carried out in the city of Jeddah about five years prior to our study, but the sample collection was not representative of the population due to the use of two malls for the recruitment of participants. In addition, they used different cut-offs to define a short and long sleep duration, defining a short sleep duration as <7 h/night, and a longer sleep duration as >7 h/night, with >8 h/night as a subgroup, and no adjustment for age was carried out, even though they reported its effect. The second Saudi study was conducted in the City of Hail in the North, measuring blood pressure only and using an electronic questionnaire to collect data on sleep duration as well as all other variables, including weight and height [65]. They reported that a longer sleep duration during the weekend only was associated with hypertension without adjusting for the effect of age. A recent meta-analysis showed that in pooled data, after adjusting for age and gender, compared with a duration of seven hours of sleep, both shorter and longer sleep duration were found to be associated with hypertension, with a sleep duration of five hours or less having the largest OR [66]. However, when gender specific data were analyzed, women with a short sleep duration (sleep time ≤ 6 h vs. 7 h) had a significantly higher risk of hypertension, while men showed an insignificant association, in a similar manner to our findings [66].

It has been reported that tobacco smoking is one of the main preventable causes of hypertension and myocardial infarction [67,68,69]. In our study there was no association between high blood pressure and smoking habits, after adjusting for age. This was similar to findings from the national study [14] Even though cigarette smoking acutely increases blood pressure, mainly through the stimulation of the sympathetic nervous system, the available data do not present clear evidence supporting a direct causal relationship between chronic smoking and elevated blood pressure [70]. This is supported by the evidence that no lower blood pressure values have been observed after chronic smoking cessation [71]. Indeed, one study reported lower blood pressure levels among smokers compared to former smokers [72], and two other studies reported increased blood pressure after smoking cessation [73,74].

However, hypertensive smokers have been reported to be more likely to develop severe forms of hypertension compared to nonsmokers [69,70]. Therefore, treating physicians usually advise their hypertensive patients to quit smoking, which might explain the lack of association between smoking and hypertension in this study, since many of the nonsmokers were former smokers.

Our study has many points of strengths in addition to a few limitations. The main strength lies in the avoiding of bias in the sample selection by recruiting participants randomly from randomly selected PHCCs representing the different geographical regions of the city, and hence the different sociodemographic classes and ethnicities. In addition, well-trained medical students were involved in data collection, using well-standardized methods in a clinical setting of the health care centers. Furthermore, the accuracy of the laboratory results was assured by performing all biochemical measurements in one accredited laboratory.

The first limitation of our study lies in its cross-sectional design, which allows only an association between studied variables, but not causation, to be suggested. Another limitation is due to the exclusion of all people with diabetes, leading to the exclusion of most people in the older age group; hence only a few individuals > 64 years of age were included.

## 5. Conclusions

In conclusion, our study showed that age, ethnicity, obesity, as well as various lifestyle factors, are associated with prehypertension and hypertension in a gender-specific manner. After adjusting for age, all measures of obesity (BMI, abdominal obesity level 2, neck circumference, elevated waist to hip, and to height ratios) were found to be associated with prehypertension and hypertension in men and women. In addition, hypertension among women only was significantly increased for those descending from central Asia, and to a lesser extent for those from southeast Asia or of mixed origin. On the other hand, high blood pressure was found to be associated with low physical activity in men, and with a sleep duration of ≤6 h and sitting for ≥4 h in women. Therefore, hypertension may be prevented in the Saudis by increasing physical activity, especially because such an increase can counter the effect of sedentary behavior according to a number of experimental studies in overweight/obese and hypertensive adults, which reported reductions in blood pressure when sitting is interrupted with intermittent light-intensity activity [75,76,77,78]. Moreover, increasing physical activity will help in reducing weight and decreasing the prevalence of obesity [79], which is another modifiable factor that has been found to be associated with hypertension in our study.

## Figures and Tables

**Table 1:** Mean and age groups of participating men and women according to their BP status.

Variable	Men					Women				
	Total	Non-Hypertensive	Prehypertensive	Hypertensive	*p*-Value	Total	Non-Hypertensive	Prehypertensive	Hypertensive	*p*-Value
	(N = 742)	(N = 280)	(N = 350)	(N = 112)		(N = 592)	(N = 361)	(N = 146)	(N = 85)	
	n; %	% (95% CI)	% (95% CI)	% (95% CI)		n; %	% (95% CI)	% (95% CI)	% (95% CI)	
Age group (years)										
(mean ± SD)	-	29.2 ± 9.0	29.9 ± 8.8	37.2 ± 14.3	*b* ***; *c* ***	-	31.4 ± 10.5	33.5 ± 12.3	40.9 ± 14.3	*a* *; *b* ***; *c* ***
<35	549; 73.9	77.5 (72.2–82.3)	76.9 (72.1–81.2)	56.3 (46.6–65.6)	*b* ***; *c* ***	354; 59.8	65.9 (60.8–70.8)	59.6 (51.2–67.6)	34.1 (24.2–45.2)	*a* *; *b* ***; *c* **
35–44	119; 16.0	17.1 (12.9–22.1)	14.0 (10.5–18.1)	19.5 (12.7–28.2)		125; 21.1	19.4 (15.4–23.9)	23.3 (16.7–31.0)	24.7 (16.0–35.3)	
45–54	42; 5.7	3.6 (1.7–6.4)	7.1 (4.7–10.4)	6.3 (2.6–12.5)		76; 12.8	12.5 (9.2–16.3)	8.9 (4.8–14.8)	21.2 (13.1–31.4)	
55–64	21; 2.8	1.1 (0.2–3.1)	1.7 (0.6–3.7)	10.7 (5.7–18.0)		30; 5.07	1.9 (0.7–4.0)	6.9 (3.3–12.2)	15.3 (8.4–24.7)	
>64	11; 1.5	0.7 (0.1–2.6)	1; 0.3 (0.01–1.6)	7.1 (3.1–13.6)		7; 1.2	0.3 (0.01–1.5)	1.4 (0.2–4.9)	4.7 (1.3–11.6)	
Proportion of people unaware of their condition	417; 56.2	NA	96.0 (93.4–97.8)	72.3 (63.1–80.4)	*c* ***	30.6	NA	93.2 (87.8–96.7)	52.9 (41.8–63.9)	*c* ***

N: total number of subjects in all subgroups; n: number of people in subgroup; Prehypertension was defined as systolic blood pressure (SBP): 120 to 139 mm Hg, and/or diastolic blood pressure (DBP) 85 to 89 mm Hg; Hypertension was defined as SBP ≥ 140, and/or DBP ≥ 90 mm Hg or taking blood pressure lowering treatment; *a* Non-hypertensive vs. Pre-hypertensive; *b* Non-hypertensive vs. hypertensive; *c* Prehypertensive vs. hypertensive; *** *p*-value > 0.001; ** *p*-value > 0.01; * *p*-value > 0.05; NA, not applicable.

**Table 2:** Association between demographic, lifestyle, vital signs, and anthropometric characteristics with prehypertension and hypertension in the male participants.

Variable	Total	Non-Hypertensive	Prehypertensive	Hypertensive	*p*-Value
	(N = 742)	(N = 280)	(N = 350)	(N = 112)	
	n; %	% (95% CI)	% (95% CI)	% (95% CI)	
**Body Mass Index (kg/m^2^)**					
(mean ± SD)	-	25.6 ± 5.2	28.0 ± 5.7	30.4 ± 6.7	*a* ***; *b* ***; *c* ***
<25	262; 35.3	47.1 (41.2–53–2)	30.6 (25.8–35.7)	20.5 (13.5–29.2)	*a* ***; *b* ***; *c* **
25–<30	275; 37.0	36.8 (31.1–42.7)	38.9 (33.7–44.2)	32.1 (23.6–41.6)	
≥30	205; 27.6	16.1 (12.0–21.0)	30.6 (25.8–35.7)	47.3 (37.8–57.0)	
**Waist Circumference (cm)**					
(mean ± SD)	-	91.5 ± 13.8	97.7 ± 14.9	104.4± 16.6	*a* ***; *b* ***; *c* ***
Normal < 94 cm	350; 47.2	60.0 (54.0–65.8)	43.7 (38.5–49.1)	25.9 (18.1–35.0)	*a* ***; *b* ***; *c* **
Abdominal obesity Level 1 ≥ 94–102 cm	172; 19.1	21.8 (17.1–27.1)	25.1 (20.7–30.0)	20.5 (13.5–29.2)	
Abdominal obesity Level 2 > 102 cm	211; 28.4	17.9 (13.6–22.9)	30.0 (25.2–35.1)	50.0 (40.4–59.6)	
**Waist to Height ratio**					
(mean ± SD)	-	0.53 ± 0.08	0.57± 0.09	0.61 ± 0.09	*a* ***; *b* ***; *c* ***
Normal ≤ 0.5	192; 25.9	36.1 (30.4–42.0)	22.0 (17.8–26.7)	12.5 (7.0–20.1)	*a* ***; *b* ***; *c* *
High > 0.5	541; 72.9	63.6 (57.6–69.2)	76.9 (72.1–81.1)	83.9 (75.8–90.2)	
**Waist to Hip ratio**					
(mean ± SD)	-	0.88 ± 0.07	0.91 ±0.08	0.93 ± 0.07	*a* ***; *b* ***; *c* *
Normal ≤ 0.95	581; 78.3	85.0 (80.3–89.0)	76.9 (72.1–81.1)	66.1 (56.5–74.8)	*a* *; *b* ***
High > 0.95	152; 20.5	14.6 (10.7–19.3)	22.0 (17.8–26.7)	30.4 (22.0–39.8)	
**Neck Circumference**					
(mean ± SD)	-	37.9 ± 3.7	40.0 ± 4.3	41.2 ± 4.3	*a* ***; *b* ***
Normal < 37	160; 21.6	32.9 (27.4–38.7)	16.0 (12.3–20.3)	10.7 (5.7–18.0)	*a* ***; *b* ***
High ≥ 37	572; 77.1	66.4 (60.6–71.9)	82.9 (78.5–86.7)	85.7 (77.8–91.6)	
**SBP (mmHg)**					
(mean ± SD)	-	110.1 ± 6.4	124.7 ± 5.6	136.9± 15.3	*a* ***; *b* ***; *c* ***
Individuals with Normal values (SBP < 120 mmHg)	295; 39.8	100.0 (98.7–100.0)	2.0 (0.8–4.1)	7.1 (3.1–13.6)	*a* ***; *b* ***; *c* **
Individuals with high values (SBP ≥ 120 mmHg)	447; 60.2	0	98.0 (95.9–99.2)	92.9 (86.4–96.9)	
DBP (mmHg)					
(mean ± SD)	-	68.2 ± 9.1	75.8 ± 8.0	88.1 ± 11.7	*a* ***; *b* ***; *c* ***
Individuals with normal values (DBP ≥ 85 mmHg)	615; 82.9	100.0 (98.7–100.0)	85.7 (81.6–89.2)	31.3 (22.8–40.7)	*a* ***; *b* ***; *c* ***
Individuals with high values (DBP ≥ 85 mmHg)	127; 17.1	0	14.3 (10.8–18.4)	68.8 (59.3–77.2)	
**Ethnic origin**					
Arabian tribes	596; 80.3	81.4 (76.4–85.8)	81.4 (77.0–85.4)	74.1 (65.0–81.9)	
African tribes	28; 3.8	2.5 (1.01–5.1)	4.3 (2.4–7.0)	5.4 (2.0–11.3)	NS
Mediterranean countries	25; 3.4	2.1 (0.8–4.6)	4.3 (2.4–7.0)	3.6 (1.0–8.9)	
Indian continent	56; 7.5	7.9 (5.0–11.7)	6.3 (4.0–9.4)	10.7 (5.7–18.0)	
Central Asia	9; 1.2	1.1 (0.2–3.1)	1.4 (0.5–3.3)	0.9 (0.02–4.9)	
South east Asia	13; 1.8	1.8 (0.6–4.1)	1.1 (0.3–2.9)	3.6 (1.0–8.9)	
Mixed	15; 2.0	3.2 (1.5–6.0)	1.1 (0.3–2.9)	1.8 (0.2–6.3)	
**Physical activity** (30 min/day–5 days/week)					
No	403; 54.3	48.6 (42.6–54.6)	56.9 (51.5–62.1)	60.7 (51.0–69.8)	*a* *; *b* *
Yes	339; 45.7	51.4 (45.4–57.4)	43.1 (37.9–48.5)	39.3 (30.2–49.0)	
**Sleep duration (h)**					
≤6	318; 42.9	42.5 (36.6–48.5)	42.9 (37.6–48.2)	43.8 (34.4–53.4)	
>6–8	381; 51.3	51.4 (45.4–57.4)	52.9 (47.5–58.2)	46.4 (37.0–56.1)	NS
>8	43; 5.8	0.6 (3.6–9.5)	42.9 (24.2–69.7)	9.8 (5.0–16.9)	
**Sitting hours/day**					
<4	97; 13.1	15.4 (11.3–20.1)	12.3 (9.0–16.2)	9.8 (5.0–16.9)	
4–5	225; 30.3	29.3 (24–35.0)	30.0 (25.2–35.1)	33.9 (25.3–43.5)	NS
6–8	258; 34.8	34.3 (28.7–40.2)	36.0 (31.0–41.3)	32.1 (23.6–41.6)	
>8	162; 21.8	21.1 (16.5–26.3)	21.7 (17.5–26.4)	24.1 (16.5–33.1)	
**Smoking Habits**					
Non smoker	463; 62.4	61.4 (55.5–67.2)	65.1 (59.9–70.1)	56.3 (46.6–65.6)	NS
Smoker	279; 37.6	38.6 (32.8–44.6)	34.9 (29.9–40.1)	43.8 (34.4–53.4)	
**Daily fruit or vegetable intake (at least one portion)**					
No	295; 39.8	36.8 (31.1–42.7)	42.9 (37.6–48.2)	37.5 (28.5–47.2)	NS
Yes	447; 60.2	63.2 (57.3–68.9)	57.1 (51.8–62.4)	62.5 (52.9–71.5)	

N: total number of subjects in all subgroups; n: number of people in subgroup; Prehypertension was defined as systolic blood pressure (SBP): 120 to 139 mm Hg, and/or diastolic blood pressure (DBP) 85 to 89 mm Hg; Hypertension was defined as SBP ≥ 140, and/or DBP ≥ 90 mm Hg or taking blood pressure lowering treatment; *a* Non-hypertensive Vs. Pre-hypertensive; *b* Non-hypertensive Vs. hypertensive; *c* Prehypertensive Vs. hypertensive; *** *p*-value > 0.001; ** *p*-value > 0.01; * *p*-value > 0.05; NS non-significant.

**Table 3:** Association between demographic, lifestyle, vital signs, and anthropometric characteristics with prehypertension and hypertension in the female participants.

Variable	Total	Non-Hypertensive	Prehypertensive	Hypertensive	*p*-Value
	(N = 592)	(N = 361)	(N = 146)	(N = 85)	
	n; %	% (95% CI)	% (95% CI)	% (95% CI)	
**Body Mass Index (kg/m^2^)**					
(mean ± SD)	-	26.0 ± 5.5	28.7 ± 6.4	30.99 ± 7.15	*a* ***; *b* ***; *c* *
<25	223; 37.7	44.0 (38.9–49.3)	32.2 (24.7–40.4)	20.0 (12.1–30.1)	*a* ***; *b* ***; *c* *
25–<30	195; 32.9	35.5 (30.5–40.6)	30.8 (23.5–39.0)	25.9 (17.0–36.5)	
≥30	174; 29.4	20.5 (16.5–25.0)	37.0 (29.2–45.4)	54.1 (43.0–65.0)	
**Waist Circumference (cm)**					
(mean ± SD)	-	84.3 ± 14.3	91.4 ±16.3	98.0 ± 16.2	*a* ***; *b* ***; *c* **
Normal < 80 cm	211; 35.6	44.0 (38.9–49.3)	28.1 (21.0–36.1)	25.9 (18.1–35.0)	*a* ***; *b* ***; *c* **
Abdominal obesity Level 1 ≥ 80–88 cm	95; 16.1	17.7 (13.9–22.1)	13.7 (8.6–20.4)	12.9 (6.6–22.0)	
Abdominal obesity Level 2 > 88 cm	270; 45.6	35.2 (30.3–40.4)	54.8 (46.4–63.0)	74.1 (63.5–83.0)	
**Waist to Height ratio**					
(mean ± SD)	-	0.53 ± 0.09	0.58 ± 0.11	0.62 ± 0.1	*a* ***; *b* ***; *c* **
Normal ≤ 0.5	194; 32.8	40.2 (35.1–45.4)	26.0 (19.1–34.0)	12.9 (6.6–22.0)	*a* **; *b* ***
High > 0.5	382; 64.5	56.8 (51.5–62.0)	70.6 (62.5–77.8)	87.1 (78.0–93.4)	
**Waist to Hip ratio**					
(mean ± SD)	-	0.82 ± 0.09	0.85 ± 0.09	0.88 ± 0.09	*a* **; *b* ***; *c* *
Normal ≤ 0.8	216; 36.5	43.5 (38.3–48.8)	30.8 (23.5–39.0)	16.5 (9.3–26.1)	*a* **; *b* ***
High > 0.8	360; 60.8	53.5 (48.2–58.7)	65.8 (57.5–73.4)	83.5 (73.9–90.7)	
**Neck Circumference**					
(mean ± SD)	-	32.7 ± 3.4	34.5 ± 5.2	35.8 ± 3.9	*a* ***; *b* ***; *c* *
Normal < 34	314; 53.0	62.1 (56.8–67.1)	43.8 (35.6–52.3)	30.6 (21.1–41.5)	*a* ***; *b* ***; *c* *
High ≥ 34	261; 44.1	34.6 (29.7–39.8)	52.7 (44.3–61.1)	69.4 (58.5–79.0)	
**SBP (mmHg)**					
(mean ± SD)	-	104.2 ± 8.2	121.9 ± 4.9	133.4 ± 18.7	*a* ***; *b* ***; *c* ***
Individuals with Normal values (SBP < 120 mmHg)	385; 65.0	100.0 (99.0–100.0)	7.5 (3.8–13.1)	15.3 (8.4–24.7)	*a* ***; *b* ***
Individuals with high values (SBP ≥ 120 mmHg)	207; 35.0	0	92.5 (86.9–96.2)	84.7 (75.3–91.6)	
**DBP (mmHg)**					
(mean ± SD)	-	65.2 ± 7.9	74.8 ± 8.8	86.5 ± 11.7	*a* ***; *b* ***; *c* ***
Individuals with normal values (DBP ≥ 85 mmHg)	513; 86.7	100.0 (99.0–100.0)	84.9 (78.1–90.3)	32.9 (23.1–44.0)	*a* ***; *b* ***; *c* ***
Individuals with high values (DBP ≥ 85 mmHg)	79; 13.3	0	15.1 (9.7–21.9)	67.1 (56.0–76.9)	
**Ethnic origin**					
Arabian tribes	429; 72.5	74.5 (69.7–78.9)	77.4 (69.8–83.9)	55.3 (44.1–66.1)	*b* **
African tribes	43; 7.3	6.7 (4.3–9.7)	8.2 (4.3–13.9)	8.2 (3.4–16.2)	
Mediterranean countries	37; 6.3	6.4 (4.1–9.4)	4.1 (1.5–8.7)	9.4 (4.2–17.7)	
Indian continent	28; 4.7	5.0 (3.0–7.8)	3.4 (1.1–7.8)	5.9 (1.9–13.2)	
Central Asia	11; 1.9	1.1 (0.3–2.8)	1.4 (0.2–4.9)	5.9 (1.9–13.2)	
South east Asia	20; 3.4	2.8 (1.3–5.0)	2.1 (0.4–5.9)	8.2 (3.4–16.2)	
Mixed	24; 4.1	3.6 (1.9–6.1)	3.4 (1.1–7.8)	7.1 (2.6–14.7)	
**Physical activity** (30 min/day–5 days/week)					
No	343; 57.9	58.5 (53.2–63.6)	58.9 (50.5–67.0)	54.1 (43.0–65.0)	NS
Yes	249; 42.1	41.6 (36.4–46.8)	41.1 (33.0–49.5)	45.9 (35.0–57.0)	
**Sleep duration (h)**					
≤6	201; 34.0	29.6 (25.0–34.6)	41.1 (33.0–49.5)	40.0 (29.5–51.2)	*a* *
>6–8	308; 52.0	54.9 (49.6–60.1)	47.3 (39.0–55.7)	48.2 (37.3–59.3)	
>8	83; 14.0	15.5 (11.9–19.7)	11.6 (6.9–18.0)	11.8 (5.8–20.6)	
**Sitting hours/day**					
<4	149; 25.2	27.7 (23.2–32.6)	18.5 (12.6–25.8)	25.9 (17.0–36.5)	NS
4–5	182; 30.7	29.4 (24.7–34.4)	32.9 (25.3–41.1)	32.9 (23.1–44.0)	
6–8	155; 26.2	26.6 (22.1–31.5)	28.8 (21.6–36.8)	20.0 (12.1–30.1)	
>8	106; 17.9	16.3 (12.7–20.6)	19.9 (13.7–27.3)	21.2 (13.1–31.4)	
**Smoking Habits**					
Non smoker	491; 82.9	82.8 (78.5–86.6)	84.3 (77.3–89.7)	81.2 (71.2–88.8)	NS
Smoker	101; 17.1	17.2 (13.4–21.5)	15.8 (10.3–22.7)	18.8 (11.2–28.8)	
**Daily fruit or vegetable intake (at least one portion)**					
No	191; 32.3	33.0 (28.1–38.1)	33.6 (26.0–41.8)	27.1 (18.0–37.8)	NS
Yes	401; 67.7	67.0 (61.9–71.9)	66.4 (58.2–74.0)	72.9 (62.2–82.0)	

N: total number of subjects in all subgroups; n: number of people in subgroup; Prehypertension was defined as systolic blood pressure (SBP): 120 to 139 mm Hg, and/or diastolic blood pressure (DBP) 85 to 89 mm Hg; Hypertension was defined as SBP ≥ 140, and/or DBP ≥ 90 mm Hg or taking blood pressure lowering treatment; *a* Non-hypertensive vs. Pre-hypertensive; *b* Non-hypertensive vs. hypertensive; *c* Prehypertensive vs. hypertensive; *** *p*-value <0.001; ** *p*-value < 0.01; * *p*-value < 0.05; NS non-significant.

**Table 4:** Unadjusted and age-adjusted odds ratio (OR), and confidence interval (CI) for anthropometric measurements and lifestyle habits covariates associated with people with prehypertension and hypertension combined.

Variable	Men		Women	
	Unadjusted OR, 95% CI	Age Adjusted OR, 95% CI	Unadjusted OR, 95% CI	Age Adjusted OR, 95% CI
**High Body Mass Index (kg/m^2^)**				
<25	Ref	Ref	Ref	Ref
25–<30	1.70 (1.20–2.39) *	1.58 (1.11–2.24)	1.31 (0.86–1.97)	1.12 (0.73–1.73)
≥30	3.61 (2.40–5.44) **	3.36 (2.22–5.09) **	3.43 (2.25–5.20) **	2.65 (1.68–4.19) **
**High Waist Circumference (cm)**				
Normal	Ref	Ref	Ref	Ref
Abdominal obesity Level 1	1.68 (1.15–2.45) *	1.58 (1.08–2.32)	1.48 (0.87–2.52)	1.33 (0.77–2.28)
Abdominal obesity Level 2	2.97 (2.03–4.35) **	2.69 (1.81–3.99) **	3.44 (2.32–5.11) **	2.76 (1.78–4.30) **
**Elevated Waist to Height ratio** > 0.5	2.26 (1.62–3.17) **	2.02 (1.42–2.87) **	2.55 (1.75–3.74) **	1.95 (1.28–2.96) *
**Elevated Waist to Hip ratio** > 0.95	1.88 (1.27–2.79) *	1.60 (1.06–2.42) *	2.30 (1.60–3.31) **	1.92 (1.31–2.80) *
**High Neck Circumference (cm)**	3.86 (1.99–4.11) **	2.65 (1.84–3.83) **	2.66 (1.88–3.75) **	2.22 (1.55–3.19) **
≥37 cm Men; ≥34 Women				
**Ethnic origin**				
Arabian tribes	Ref	Ref	Ref	Ref
African tribes	1.86 (0.78–4.44)	1.72 (0.71–4.13)	1.33 (0.71–2.51)	1.34 (0.71–2.55)
Mediterranean countries	1.96 (0.77–4.99)	1.95 (0.76–4.96)	1.02 (0.51–2.05)	0.89 (0.43–1.81)
Indian continent	0.96 (0.55–1.68)	0.92 (0.52–1.61)	0.93 (0.42–2.07)	1.02 (0.45–2.29)
Central Asia	1.24 (0.31–5.00)	1.52 (0.38–6.19)	2.94 (0.85–10.21)	3.38 (0.96–11.87)
Southeast Asia	0.99 (0.32–3.07)	1.01 (0.32–3.17)	1.68 (0.69–4.13)	1.48 (0.59–3.67)
Mixed	0.41 (0.15–1.18)	0.47 (0.16–1.34)	1.42 (0.62–3.25)	1.48 (0.63–3.46)
**Low Physical activity**	1.45 (1.08–1.95) *	1.41 (1.04–1.90) *	0.95 (0.68–1.32)	0.89 (0.63–1.25)
(<30 min/day–5 days/week)				
**Sleep duration (h)**				
>6–8	Ref	Ref	Ref	Ref
≤6	1.02 (0.75–1.38)	0.99 (0.73–1.35)	1.58 (1.10–2.27) **	1.48 (1.02–2.14) *
>8	0.82 (0.49–1.77)	0.94 (0.49–1.80)	0.87 (0.52–1.45)	1.01 (0.59–1.71)
**Sitting hours/day**				
<4	Ref	Ref	Ref	Ref
4–5	1.39 (0.86–2.25)	1.33 (0.81–2.16)	1.46 (0.93–2.29)	1.64 (1.03–2.61) *
6–8	1.34 (0.84–2.16)	1.34 (0.83–2.15)	1.25 (0.78–2.01)	1.74 (1.05–2.72) *
>8	1.39 (0.83–2.32)	1.37 (0.82–2.29)	1.63 (0.97–2.72)	1.98 (1.16–3.38) *
**Smoking Habits**	0.94 (0.69–1.27)	0.97 (0.71–1.31)	0.98 (0.63–1.52)	1.00 (0.65–1.58)

* *p*-value < 0.05; ** *p*-value < 0.001; High Waist Circumference (cm); Normal ≤ 94 cm Men; ≤80 cm Women; Abdominal obesity Level 1 > 94–102 cm Men; >80–88 cm women; Abdominal obesity Level 2 > 102 cm Men; >88 cm Women.

**Table 5:** Unadjusted and age-adjusted odds ratio (OR), and confidence interval (CI) for anthropometric measurements and lifestyle habits covariates associated with hypertension.

Variable	Men		Women	
	Unadjusted OR, 95% CI	Age Adjusted OR, 95% CI	Unadjusted OR, 95% CI	Age Adjusted OR, 95% CI
**High Body Mass Index (kg/m^2^)**				
<25	Ref	Ref	Ref	Ref
25–<30	2.00 (1.12–3.59) *	1.55 (0.84–2.85)	1.56 (0.80–3.01)	1.15 (0.57–2.32)
≥30	6.76 (3.73–12.3) **	5.38 (2.90–9.99) **	6.00 (3.29–10.9) **	3.18 (1.59–6.35) **
**High Waist Circumference (cm)**				
Normal	Ref	Ref	Ref	Ref
Abdominal obesity Level 1	2.18 (1.17–4.06) *	1.64 (0.85–3.13)	2.48 (1.03–6.02) *	1.84 (0.74–4.59)
Abdominal obesity Level 2	6.49 (3.75–11.23) **	4.54 (2.54–8.07) **	7.17 (1.03–14.18) **	4.09 (1.94–8.65) **
**Elevated Waist to Height ratio** > 0.5	3.81 (2.07–7.03) **	2.44 (1.28–4.67) **	4.76 (2.44–9.28) **	2.62 (1.27–5.41) **
**Elevated Waist to Hip ratio** > 0.95	2.67 (1.58–4.51) *	1.59 (0.89–2.85)	4.13 (2.24–7.60) **	3.03 (1.61–5.71) **
**High Neck Circumference (cm)**	3.56 (1.89–6.69) **	2.73 (1.42–5.28) **	3.92 (2.35–6.53) **	2.78 (1.63–4.76) **
≥37 cm Men; ≥34 Women				
**Ethnic origin**				
Arabian tribes	Ref	Ref	Ref	Ref
African tribes	2.36 (0.77–7.20)	2.08 (0.64–6.77)	1.67 (0.68–4.09)	1.92 (0.75–4.95)
Mediterranean countries	1.83 (0.50–6.65)	1.90 (0.49–7.41)	1.99 (0.84–4.71)	1.69 (0.65–4.41)
Indian continent	1.50 (0.71–3.16)	1.53 (0.70–3.33)	1.59 (0.56–4.49)	2.38 (0.80–7.11)
Central Asia	0.92 (0.09–8.93)	1.59 (0.16–15.81)	7.15 (1.85–27.62) *	11.54 (2.76–48.18) **
South east Asia	2.20 (0.58–8.38)	2.48 (0.61–10.06)	4.00 (1.45–11.05) *	3.51 (1.22–10.13) *
Mixed	0.61 (0.13–2.88)	0.89 (0.18–4.37)	2.64 (0.96–7.30)	3.24 (1.04–10.08) *
**Low Physical activity** (<30 min/day–5 days/week)	1.64 (1.05–2.56) *	1.51 (0.95–2.42)	0.84 (0.52–1.35)	0.70 (0.42–1.17)
**Sleep duration (h)**				
>6–8	Ref	Ref	Ref	Ref
≤6	1.14 (0.72–1.81)	1.00 (0.62–1.64)	1.54 (0.92–2.56)	1.25 (0.73–2.16)
>8	1.64 (0.79–4.08)	1.76 (0.73–4.23)	0.86 (0.41–1.83)	1.15 (0.52–2.52)
**Sitting hours/day**				
<4	Ref	Ref	Ref	Ref
4–5	1.81 (0.84–3.90)	1.67 (0.74–3.75)	1.20 (0.65–2.24)	1.46 (0.76–2.81)
6–8	1.47 (0.68–3.15)	1.71 (0.76–3.84)	0.81 (0.40–1.61)	1.12 (0.54–2.33)
>8	1.79 (0.80–3.99)	1.90 (0.81–4.42)	1.39 (0.69–2.80)	2.01 (0.95–4.28)
**Smoking Habits**	1.24 (0.79–1.93)	1.54 (0.96–2.48)	1.12 (0.61–2.06)	1.20 (0.63–2.278)

* *p*-Value < 0.05; ** *p*-Value < 0.001; High Waist Circumference (cm); Normal ≤ 94 cm Men; ≤80 cm Women; Abdominal obesity Level 1 > 94–102 cm Men; >80–88 cm women; Abdominal obesity Level 2 > 102 cm Men; >88 cm Women.

## Data Availability

The datasets analyzed for this study can be found at king Abdulaziz university repository at http://www.kau.edu.sa/GetFile.aspx?id=306527&fn=RS (accessed on 26 August 2021).
